# Supplementary figures and images for: The Role of Psychological Factors and Vaccine Conspiracy Beliefs in Influenza Vaccine Hesitancy and Uptake among Jordanian Healthcare Workers during the COVID-19 Pandemic
Source: Vaccines (Basel). 2022 Aug 19;10(8):1355. doi: 10.3390/vaccines10081355 (PMC9413675; doi:10.3390/vaccines10081355)

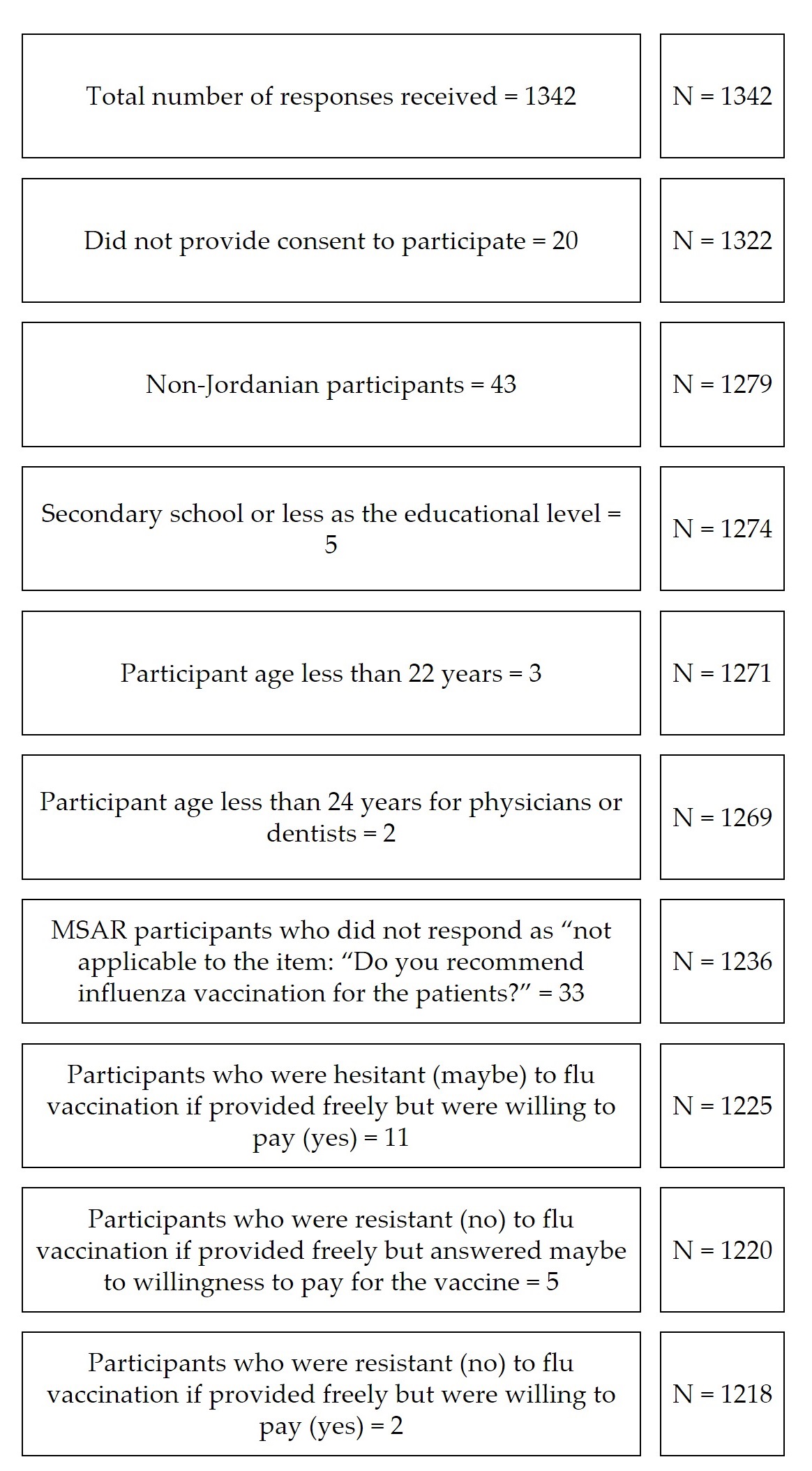

Supplement: Supplementary file 1 [file vaccines-10-01355-s001.zip › Supplementary-File.jpg]
